# Bronchodilator Delivery via High-Flow Nasal Cannula: A Randomized Controlled Trial to Compare the Effects of Gas Flows

**DOI:** 10.3390/pharmaceutics13101655

**Published:** 2021-10-11

**Authors:** Jie Li, Yibing Chen, Stephan Ehrmann, Jie Wu, Lixin Xie, James B Fink

**Affiliations:** 1 Rush University Medical Center, Department of Cardiopulmonary Sciences, Division of Respiratory Care, Chicago, IL 60612, USA; Jie_Li@rush.edu (J.L.); James_Fink@rush.edu (J.B.F.); 2 Pulmonary Function Test Lab, Department of Respiratory and Critical Care Medicine, People’s Liberation Army General Hospital, 28 Fuxing Road (Wukesong), Beijing 100083, China; chenyibing1128@126.com (Y.C.); wujie771117@hotmail.com (J.W.); 3 CHRU Tours, Médecine Intensive Réanimation, CIC INSERM 1415, CRICS-TriggerSEP FCRIN Research Network, 37000 Tours, France; stephan.ehrmann@univ-tours.fr; 4 INSERM, Centre D’étude des Pathologies Respiratoires, U1100, Université de Tours, 37000 Tours, France; 5 Aerogen Pharma Corp, San Mateo, CA 94402, USA

**Keywords:** high-flow nasal cannula, aerosol therapy, asthma, chronic obstructive pulmonary disease

## Abstract

(1) Background: Aerosol delivery via high-flow nasal cannula (HFNC) has attracted increasing clinical interest. In vitro studies report that the ratio of HFNC gas flow to patient inspiratory flow (GF:IF) is a key factor in the efficiency of trans-nasal aerosol delivery. (2) Methods: In a randomized controlled trial, patients with a history of COPD or asthma and documented positive responses to inhaled bronchodilators in an outpatient pulmonary function laboratory were recruited. Subjects were randomized to receive inhalation at gas flow ratio settings of: GF:IF = 0.5, GF:IF = 1.0, or GF = 50 L/min. Subjects were assigned to inhale saline (control) followed by salbutamol via HFNC with cumulative doses of 0.5 mg, 1.5 mg, 3.5 mg, and 7.5 mg. Spirometry was performed at baseline and 10–12 min after each inhalation. (3) Results: 75 subjects (49 asthma and 26 COPD) demonstrating bronchodilator response were enrolled. Per the robust ATS/ERS criteria no difference was observed between flows, however using the criteria of post-bronchodilator forced expiratory volume in the first second (FEV_1_) reaching the screening post-bronchodilator FEV_1_ with salbutamol, a higher percentage of subjects receiving GF:IF = 0.5 met the criteria at a cumulative dose of 1.5 mg than those receiving GF:IF = 1.0, and GF = 50 L/min (64% vs. 29% vs. 27%, respectively, *p* = 0.011). Similarly at 3.5 mg (88% vs. 54% vs. 46%, respectively, *p* = 0.005). The effective dose at GF:IF = 0.5 was 1.5 mg while for GF = 50 L/min it was 3.5 mg. (4) Conclusions: During salbutamol delivery via HFNC, cumulative doses of 1.5 mg to 3.5 mg resulted in effective bronchodilation. Applying the robust ATS/ERS criteria no difference was observed between the flows, however using the more sensitive criteria of subjects reaching post screening FEV_1_ to salbutamol via HFNC, a higher number of subjects responded to the doses of 0.5 mg and 1.5 mg when HFNC gas flow was set at 50% of patient peak inspiratory flow.

## 1. Introduction

Due to evidence of improving oxygenation and avoiding intubation for patients with hypoxemic respiratory failure [1,2,3,4], high-flow nasal cannula (HFNC) has been broadly utilized [5]. This is attributed to gas flow that meets or exceeds the patient’s inspiratory flow, resulting in a constant fraction of inspired oxygen (F_I_O_2_) and some amount of positive airway pressure [3]. While the high gas flow washes out the dead space, work of breathing may decrease and carbon dioxide clearance may increase, the utilization of HFNC is expanded to patients with hypercapnic respiratory failure, including the use for acute exacerbation of chronic obstructive pulmonary disease (COPD) or asthma [6,7,8,9], for facilitating weaning from invasive ventilation [10], and for improving quality of life with domiciliary long-term use [11].

During HFNC therapy, patients may require inhaled bronchodilator or other medical aerosol therapy [5]. Placing the nebulizer over HFNC was shown to deliver little to no drug to patients [12] while discontinuing HFNC to use conventional aerosol devices may sacrifice the benefits of HFNC [13]. Similarly, placing metered-dose inhalers (MDI) and spacer over HFNC [14] or in-line with HFNC [15] are found to deliver only 1/6 to 1/3 of the inhaled dose delivered without HFNC. Both in vitro and in vivo testing have provided varying estimates of trans-nasal HFNC aerosol delivery efficiency at different flows and breathing patterns [16,17,18,19,20,21,22,23], leaving questions as to what inhaled drug doses might be used.

Short-acting bronchodilators such as albuterol are commonly used to treat patients with COPD and asthma, both at home and in the acute care setting. The standard for identifying responses to short-acting bronchodilators is based on spirometry results. We previously reported a doubling dose titration study in subjects with stable mild to moderate COPD or asthma, to identify the cumulative dose required via HFNC to elicit standard bronchodilator response [19]. However, a single gas flow was used (15–20 L/min), which is lower than the 40–60 L/min commonly administered via HFNC in the emergency department or intensive care unit (ICU) [1,2,3,4,5] and potentially associated with reduced aerosol delivery [16,17,18].

Indeed, in vitro, the ratio of HFNC gas flow to patient peak inspiratory flow (GF:IF) was found to play a key role in the trans-nasal aerosol delivery efficiency. When aerosolized medication is delivered via HFNC, gas flow higher than patient inspiratory flow causes waste of medication and creates turbulent flows in the airways, resulting in reduced aerosol delivery to the lung [13]. The inhaled dose distal to the trachea increases as the ratio decreases, with efficiency peaking at HFNC gas flow settings around 50% of patient inspiratory flow (GF:IF = 0.5) [16]. In vitro, the inhaled dose with GF:IF = 0.5 was observed to be 2–4 folds higher than that with higher gas flows, however, clinical evidence is lacking [16]. This finding raises the question of how nominal doses loaded in the nebulizer might be adjusted to elicit a patient response to aerosol bronchodilators administered with different gas flow settings.

To better understand how HFNC flow impacts inhaled bronchodilator dose required to induce bronchodilation and confirm that GF:IF ratio may reduce dosing requirements, we conducted a randomized controlled trial to investigate the minimally effective inhaled bronchodilator dose at various GF:IF ratios. Considering the efficient aerosol delivery at GF:IF = 0.5 via HFNC, we proposed that more patients in the group of GF:IF = 0.5 would respond to bronchodilator at low doses (0.5 mg and 1.5 mg) via HFNC.

## 2. Materials and Methods

This study was approved by the ethic committees of the People’s Liberation Army General Hospital, Beijing, China (No.S2018-200-02, approved on 29 November 2018) and Rush University, Chicago, IL, US (No.19041201-IRB01, approved on 10 July 2019). It was registered with ClinicalTrials.gov, accessed on 10 October 2021 (NCT03739359). The study protocol was also published [24].

### 2.1. Study Population

Stable patients with COPD or asthma with positive results in the standard bronchodilator test per ATS/ERS (American Thoracic Society/European Respiratory Society) standards were recruited at an outpatient pulmonary function test (PFT) laboratory at People’s Liberation Army General Hospital. Positive response for the bronchodilator test was defined as the forced expiratory volume in the first second (FEV_1_) increased by ≥12% with an absolute change of ≥200 mL from baseline [25], after inhaling 400 mcg salbutamol (Ventolin, GSK, UK) from a metered-dose inhaler (MDI) with a valved holding chamber (VHC, OptiChamber Diamond, Philips, Parsippany, NJ, USA).

Subjects were excluded if meeting any of the following criteria: age ≥ 90 years; pregnancy; pulmonary exacerbation within two weeks; lack of informed consent; inability to complete the follow-up spirometry after each bronchodilator inhalation; resting heart rate > 100 bpm; resting systolic blood pressure > 160 mmHg or diastolic blood pressure > 110 mmHg.

### 2.2. Study Procedures

Following qualifying screening spirometry, and a minimum of 24 h wash out period, subjects returned to the PFT laboratory to participate in the study. Bronchodilator treatment was withheld for required periods prior to study [24,25]. After the consent form was signed, subjects were randomized (sequentially numbered, sealed, opaque envelopes containing the treatment assignment) to three HFNC gas flows (50 L/min, GF:IF = 1.0, and GF:IF = 0.5). The randomization was stratified by disease (COPD or asthma) with a block size of six. The PFT technician who performed spirometry tests was blinded for the randomization. To ensure the quality of the test, all the tests were performed by the same PFT technician using the same calibrated spirometer.

Peak inspiratory flow was measured during quiet tidal breathing prior to forced vital capacity measurement during baseline spirometry, then subjects were instructed to inhale 0.9% normal saline (Siyao Ltd., Shijiazhuang, China) (2 mL) followed by salbutamol at an escalating doubling dose sequence (0.5 mg, 1.0 mg, 2.0 mg and 4.0 mg diluted in a constant 2 mL volume) via a vibrating mesh nebulizer (VMN, Aerogen Solo, Aerogen Ltd., Galway, Ireland), which was placed at the inlet of the humidifier chamber (MR850, Fisher & Paykel Healthcare, Auckland, New Zealand) of the HFNC circuit (Fisher & Paykel Healthcare), VMN was placed on the dry side of the humidifier due to the higher inhaled dose and the lower condensate deposited in the circuit compared to placement close to the patient [26]. Nasal cannula size (Fisher & Paykel Healthcare, Auckland, New Zealand) was chosen as less than 50% of the diameters of subjects’ nostrils. Nebulization duration ranged from 6 to 8 min to administer the 2-mL of drug volume, and nebulization was administered at an interval of ~20 min. During nebulization, subjects were instructed to breathe via the nose with the mouth closed. The assigned gas flow settings were confirmed by a mass flowmeter (TSI 4040, TSI Incorporated, Shoreview, Minnesota, USA) [24]. HFNC was removed after nebulization was completed. After 10–12 min rest, subjects repeated the forced vital capacity test. Inhalation was terminated if adverse events including tachycardia (a resting heart rate > 100 bpm), tremor, irregular heart rhythm, blood pressure (either systolic or diastolic) increase > 20% were observed or headache was reported.

### 2.3. Outcomes

The primary outcome was the rate of bronchodilation response with each of the three gas flows at each cumulative salbutamol dose. Positive response was determined by meeting any of the following criteria: (1) ATS/ERS criteria of positive bronchodilation response (see above) [25,27]; (2) absolute value of FEV_1_ post-dose inhalation via HFNC ≥ post-bronchodilator levels exhibited during screening with MDI + VHC [19]. The secondary outcome was the cumulative dose of salbutamol required with each HFNC flow setting inducing a positive bronchodilation response.

### 2.4. Sample Size Calculation

This study was a superiority study. With α level of 0.05, power (1−ß) of 80% and assuming 80% of subjects would respond to salbutamol at the cumulative dose of 1.5 mg with GF:IF = 0.5, compared to 40% with HFNC flow of 50 L/min, 25 subjects in each group and 75 in total needed to be included [24].

### 2.5. Data Collection

Demographic information (age, gender, height, weight, race, smoking history, diagnosis), baseline parameters during tidal breathing (tidal volume and peak inspiratory flow), and spirometry results before and after inhaling saline and salbutamol at each dose were recorded.

### 2.6. Statistical Analysis

Kolmogorov–Smirnov test was performed to evaluate the normality of distribution for continuous variables, which were presented as mean ± standard derivation (SD) or median and interquartile range (IQR) accordingly. One-way analysis of covariance (ANCOVA) was conducted to determine the difference among the three flow groups for changes in spirometry results, controlling for baseline variables. ANOVA was used to compare baseline variables among three flow conditions. Repeated measures ANOVA analysis was used to compare the differences in FEV_1_ increase with the different escalating bronchodilator doses among subjects receiving the same HFNC flow setting. Categorical variables were expressed as percentage and analyzed by Chi-square test. A two-sided *p*-value of <0.05 was considered statistically significant. Data analysis was performed with SPSS software (SPSS 23.0; IBM, Armonk, New York, NY, USA).

## 3. Results

**Figure 1:**
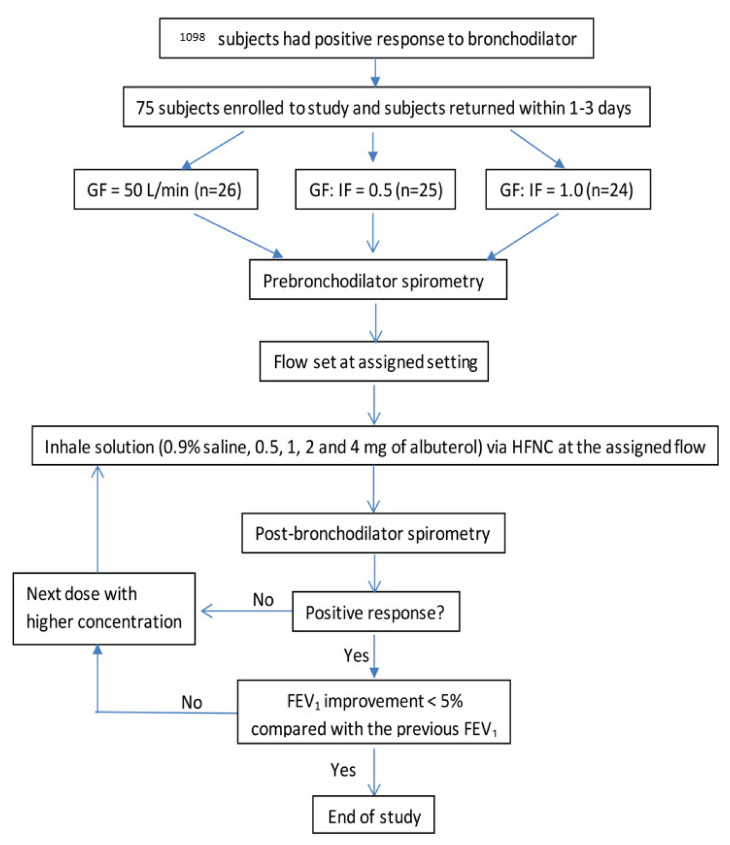
Study flowchart. GF, gas flow; IF, patient inspiratory flow; HFNC, high-flow nasal cannula; FEV_1_, forced expiratory volume in the first second.

From 7 February 2019 to 12 November 2019, 1098 patients demonstrating positive responses to bronchodilators in the PFT lab were screened. Most of the patients were excluded, due to: (1) The screening bronchodilator test was performed by other technicians or using another spirometer; (2) The patients were unavailable to return to the PFT lab to participate in the study on a separate day. Finally, 75 subjects were recruited with 25, 24 and 26 subjects assigned to receive HFNC flows of GF:IF = 0.5, GF:IF = 1.0 and GF = 50 L/min, respectively (Figure 1). A total of 49 subjects had asthma while 26 had COPD. Forty-eight (64%) subjects were male and 33 (44%) had a smoking history. No significant differences in age, gender, height, weight, pulmonary disease (asthma or COPD), smoking history, tidal volume and inspiratory flow were observed among the three groups (Table 1). No adverse events were reported.

### 3.1. Bronchodilation Responses after Inhaling Salbutamol via HFNC

All recruited subjects completed testing; results are shown in Table 2. Using the ATS/ERS criteria for positive bronchodilation response [25,27], 44% of subjects receiving GF:IF = 0.5 met the criteria after inhaling the initial salbutamol dose of 0.5 mg compared to 25% and 27% of patients receiving GF:IF = 1.0 and GF = 50 L/min (*p* = 0.286), respectively. After receiving the higher 1.5 mg dose, 64% of patients responded with the GF:IF = 0.5, a proportion similar to those receiving GF:IF = 1.0 and GF = 50 L/m (58% and 42%, respectively, *p* = 0.271). In contrast, applying the criteria of post-bronchodilator FEV_1_ via HFNC return to screening post-bronchodilator level [19], a higher percentage of subjects receiving GF:IF = 0.5 met the criteria at the cumulative dose of 1.5 mg than those receiving GF:IF = 1.0 and GF = 50 L/min (64% vs. 29% vs. 27%, *p* = 0.011), and of 3.5 mg (88% vs. 54% vs. 46%, respectively, *p* = 0.005). A higher percentage of subjects receiving GF:IF = 0.5 met both criteria at the cumulative dose of 1.5 mg than the two other flows.

### 3.2. FEV_1_ and FVC Changes after Inhaling Salbutamol via MDI + VHC and via HFNC at Different Doses

Table 3 shows the changes of FEV_1_ and FVC after inhaling salbutamol via MDI with VHC and the changes of FEV_1_ after inhaling saline and salbutamol at different doses via HFNC.

**Figure 2:**
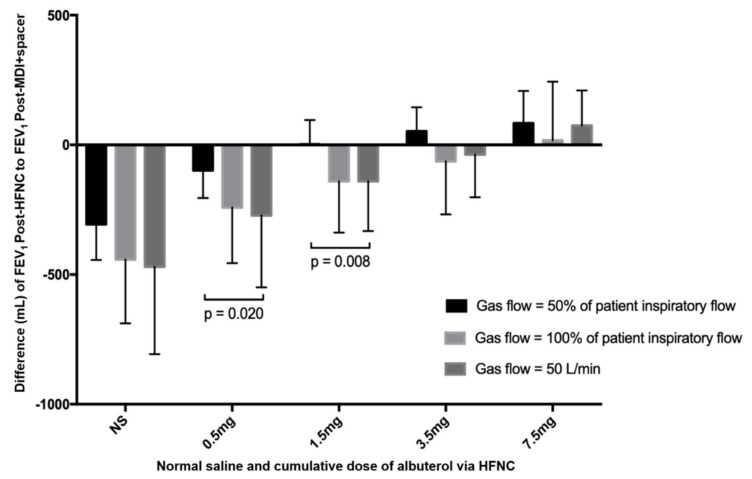
The difference of post-screening FEV_1_ and FEV_1_ after inhaling saline and salbutamol via HFNC at different nominal doses in three groups. FEV_1_ post-MDI + VHC was deemed the highest observed FEV_1_ for individual patients. Using the difference between the post-screening FEV_1_ and each FEV_1_ after inhaling saline and salbutamol via HFNC at different nominal doses to compare among three groups (ANOVA test), no significant difference was found after inhaling saline. While at the cumulative doses of 0.5 mg and 1.5 mg, the difference from post-screening FEV_1_ in the group of GF:IF = 0.5 was smaller than the other two groups, this difference became nonsignificant at the cumulative doses of 3.5 mg and 7.5 mg. Scheme 0. had a smaller FEV_1_ difference than GF = 50 L/min after inhaling salbutamol of 0.5 mg (*p* = 0.023) and 1.5 mg (*p* = 0.018) while no significant differences were found between GF:IF = 1.0 and GF = 50 L/min. FEV_1_, forced expiratory volume in the first second; HFNC, high-flow nasal cannula; MDI, metered dose inhaler; VHC, valved holding chamber; GF, gas flow; IF, inspiratory flow.

Since the inhalation of 400 mcg salbutamol via MDI + VHC during screening is the standard dose to elicit validated standard bronchodilation effect, the screening post-bronchodilator FEV_1_ was assumed to represent the validated standard FEV_1_ target that each subject achieved during screening [19,24] Using the screening post-bronchodilator FEV_1_ to calculate the difference between previously observed FEV_1_ and post-bronchodilator FEV_1_ at each dose via HFNC, the difference was smaller in subjects receiving GF:IF = 0.5 than those receiving GF:IF = 1.0 and GF = 50 L/min at the cumulative dose of 0.5 mg (−98 ± 107 vs. −241 ± 215 vs. −272 ± 277 mL, *p* = 0.020) and 1.5 mg (2 ± 94 vs. −140 ± 198 vs. −140 ± 192 mL, *p* = 0.008) (Figure 2).

**Figure 3:**
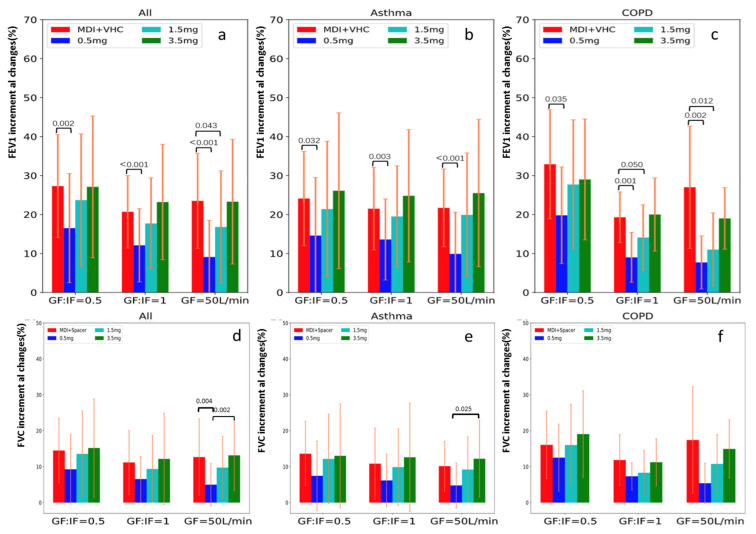
FEV_1_ and FVC improvement after inhaling salbutamol during screening (MDI + VHC) and via HFNC at different nominal doses in three groups. Using the FEV_1_ improvement after inhaling salbutamol at each cumulative dose to compare that with screening (ANCOVA test), screening FEV_1_ post-salbutamol improvement was higher than FEV_1_ improvement after inhaling salbutamol at 0.5 mg via HFNC in all three groups, however, this difference became non-significant at the cumulative dose of 1.5 mg via HFNC, except for those receiving GF = 50 L/min, the difference became non-significant at the cumulative dose of 3.5 mg (**a**). For asthma subjects, the difference of FEV1 improvement from screening became non-significant at the cumulative dose of 1.5 mg for all three flows (**b**). In contrast, for COPD subjects, post-bronchodilator FEV1 improvement was similar at 1.5 mg only with flow of GF:IF = 0.5, the differences became insignificant at 3.5 mg with flows of GF:IF = 1.0 and GF = 50 L/min (**c**). Compared to FVC improvement salbutamol via MDI + VHC during screening, no significant differences of FVC improvement in the inhalation of all doses of salbutamol via HFNC, except for the lower improvement of FVC after inhaling 0.5 mg of salbutamol via HFNC in the group of GF = 50 L/min (*p* = 0.004) (**d**). Similar responses in FVC for asthma (**e**) and COPD (**f**) subjects. HFNC, high-flow nasal cannula; FEV_1_, forced expiratory volume in the first second; MDI, metered dose inhaler; VHC, valved holding chamber; GF, gas flow; IF, inspiratory flow; COPD, chronic obstructive pulmonary disease; FVC, forced vital capacity.

Compared to the FEV_1_ improvement with MDI + VHC at screening, the FEV_1_ improvement with salbutamol via HFNC at 0.5 mg was lower in all three flow groups, however, this difference became nonsignificant at the cumulative dose of 1.5 mg with GF:IF = 0.5 and 1.0, and at the cumulative dose of 3.5 mg with GF = 50 L/min (Figure 3a). Thus the minimally effective dose for the both GF:IF = 0.5 and GF:IF = 1.0 was 1.5 mg, while 3.5 mg for GF = 50 L/min.

Compared to the FVC improvement with MDI + VHC at screening, the FVC improvement was not significantly different with salbutamol via HFNC at all the doses (0.5 mg, 1.5 mg and 3.5 mg) in the three flow groups, except it was lower in the group of GF = 50 L/min at 0.5 mg (Figure 3d).

### 3.3. Other Spirometry Results of Inhaling Salbutamol at Different Doses

**Figure 4:**
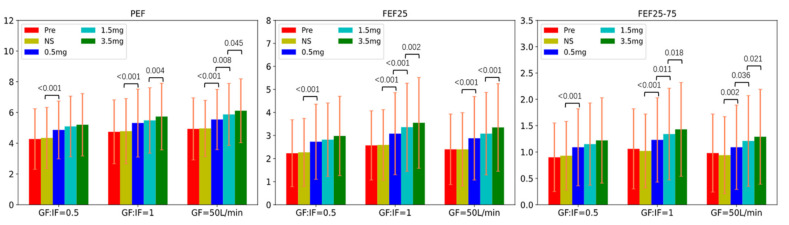
The results of PEF, FEF_25_, and FEF_25–75_ after inhaling saline and salbutamol via HFNC at different nominal doses in three groups. In the group of GF:IF = 0.5, all the spirometry results were higher after inhaling salbutamol at 0.5 mg, compared to that with saline inhalation. However, in the groups of GF:IF = 1.0 and GF = 50 L/min, PEF, FEF_25_, FEF_25–75_ continued increasing after inhaling salbutamol via HFNC at the cumulative doses of 0.5 mg, 1.5 mg and 3.5 mg. HFNC, high-flow nasal cannula; GF, gas flow; IF, inspiratory flow; PEF, peak expiratory flow; FEF_25_, forced expiratory flow at 25% of forced vital capacity; FEF_25–75_, forced expiratory flow at 25% to 75% of forced vital capacity.

After inhaling salbutamol via HFNC at 0.5 mg, subjects’ PEF, FEF_25_, and FEF_25–75_ significantly increased in all three groups, compared to inhaling saline (Figure 4). However, these variables did not change significantly with the cumulative dose of 1.5 mg with GF:IF = 0.5, in contrast to improvement with both GF:IF = 1.0 and GF = 50 L/min.

### 3.4. The Differences between Asthma and COPD Subjects at the Three HFNC Flows

For subjects with asthma, the effective dose was 1.5 mg for all three flows (Figure 3b). However, more subjects receiving GF:IF = 0.5 had their post-HFNC FEV_1_ return to the screen post-salbutamol FEV_1_ than the other subjects at cumulative doses of 1.5 mg (*p* = 0.047) and 3.5 mg (*p* = 0.002) (Table S1). For subjects with COPD, the effective dose was 1.5 mg for group of GF:IF = 0.5, while 3.5 mg for groups of GF:IF = 1.0 and GF = 50 L/min (Figure 3c). Moreover, FEV_1_ improvement at 0.5 mg was higher with GF:IF = 0.5 than the other flows (*p* = 0.040) (Table S2).

## 4. Discussion

This is the first randomized controlled trial to compare the effects of HFNC gas flow and patient peak inspiratory flows on response to transnasal bronchodilator delivery. We found that subjects receiving GF:IF = 0.5 responded to a lower cumulative doses than subjects receiving GF:IF = 1.0 and GF = 50 L/min. The effective dose to generate responses similar to baseline screening was 1.5 mg with GF:IF = 0.5 versus 3.5 mg when receiving GF = 50 L/min. These findings are consistent with our previous in vitro reports that aerosol delivery efficiency increased as GF:IF decreased to 0.5 [16].

The goal of this study was not to promote the use of HFNC for aerosol delivery to mild and moderate patients with COPD or asthma but to identify a dose level that provided a similar bronchodilator response in this patient population as the label dose. The label dose of albuterol and most other inhaled medications is mainly determined based on clinical trials performed on stable subjects with mild and moderate disease [28,29]. Consequently, this study was designed to identify an equivalent dose to achieve bronchodilator response when administering trans-nasal salbutamol via HFNC. During exacerbation, patients with COPD or asthma may require higher than the standard label salbutamol doses for treatment in the emergency room and ICU. Therefore, our reported dose levels to achieve bronchodilator response should be viewed as a starting dose. Additionally, this study validated the critical role of GF:IF concerning the efficiency of aerosol delivery via HFNC, which provides the supporting evidence to change clinical practice to titrate HFNC gas flow when aerosolized medication is delivered [22].

In a previous prospective study, of 42 stable COPD or asthma patients with similar screening criteria, 69% met ATS/ERS positive response criteria after inhaling a cumulative salbutamol dose of 1.5 mg via HFNC with the flow set at 15–20 L/min [19]. This is consistent with the 64% response at 1.5 mg in subjects receiving GF:IF = 0.5 representing a mean HFNC flow of 18.7 ± 3.9 L/min.

Among subjects receiving GF:IF = 0.5, baseline FEV_1_ prior to nebulization via HFNC (pre-HFNC) was higher than their own screening baseline. In contrast, baseline FEV_1_ pre-HFNC was lower than screening baseline in the other two groups, which required less improvement during nebulization via HFNC to meet the ATS/ERS positive response criteria than for subjects receiving GF:IF = 0.5. We previously identified this issue as a potential cause of bias based on our prior study [19] and added to the protocol identifying when FEV_1_ reached or exceeded the screening post bronchodilation reference value [24].

Using the ATS/ERS positive response criteria, we did not find significant differences in responders among the three flows tested. However, using the other criteria, the effective dose was 1.5 mg with GF:IF = 0.5, and 3.5 mg with GF = 50 L/min. Additionally, in subjects receiving GF:IF = 0.5, the spirometry results of PEF, FEF_25_, FEF_25–75_ stopped increasing after inhaling 0.5 mg salbutamol via HFNC. In contrast, the groups receiving GF:IF = 1.0 and GF = 50 L/min had significant improvements with increased dosing increments. (Figure 4).

The effective dose for subjects with asthma was 1.5 mg for all three flows, while the effective dose for COPD subjects was 1.5 mg with GF:IF = 0.5, and 3.5 mg for both GF:IF = 1.0 and GF = 50 L/min (Tables S1 and S2). This difference might be explained by the lower nominal dose needed to elicit an effective beta-agonist response for asthma subjects than COPD subjects. Fishwick and colleagues found 50 mcg of salbutamol via dry powder inhaler was able to achieve similar bronchodilation effects as 400 mcg in asthma subjects (FEV_1_ of 2.79 vs. 2.84 L) [28], while COPD patients’ FEV_1_ increased as the dose of salbutamol increased from 100 to 800 mcg [29]. In our study, COPD subjects receiving GF:IF = 0.5 required a lower cumulative dose to return FEV_1_ to screening levels can be explained in part by the higher trans-nasal delivery efficiency of aerosol at the lower flow [16,17,18,22].

Overall, these findings suggest that salbutamol dose of 1.5 mg to 3.5 mg provided effective doses depending on the HFNC flow applied, with little to no severe adverse events. Depending on the jurisdiction, standard salbutamol doses vary from 2.5 mg to 5.0 mg. A label dose of 5.0 mg should be sufficient for all stable patients receiving HFNC in the range of flows studied. As we only compared doses of 1.5 mg and 3.5 mg, it is unclear whether a unit dose of 2.5 mg would be sufficient as an effective dose at the higher flows studied. Future studies are needed to investigate if 2.5 mg is effective to elicit bronchodilation response at HFNC gas flow higher or equal to patient inspiratory flow, particularly among COPD subjects.

This is also the first study to assess the inspiratory flow for adult subjects with stable asthma and COPD before administration of HFNC. These subjects were not in acute distress or exacerbation phase in which HFNC might be more commonly utilized. Our findings of the average subject inspiratory flow of 35 L/min provides general guidance that 35–40 L/min should be the minimal flow via HFNC to avoid air entrainment in adults. Currently, no commercially available device can be used to monitor patient inspiratory flow breath-by-breath, our study provides practical suggestions on HFNC gas flow settings during trans-nasal aerosol delivery, titrating flow to 15–20 L/min for stable subjects and 25–30 L/min for subjects with distressed breathing could increase the delivery efficiency [13]. Notably, reducing flow to optimize aerosol delivery might cause desaturation and increase work of breathing, for subjects who rely on high gas flow and high oxygen concentration. For these patients, administration of small volumes of the solution may reduce dosing time to shorten the periods of flow reduction [13,30]. If the reduced flow is not tolerated, or long-term continuous inhalation is needed, a higher nominal dose might be necessary.

### Limitations

The requirement to perform repeated forced expiratory maneuvers limited us to conduct the study among subjects with stable asthma and COPD. As the utilization of HFNC has been expanded to stable COPD subjects [11], this population may more directly benefit from our results. However, the primary indication of HFNC remains for patients with acute respiratory failure, whose breathing patterns and airway response might be different from stable subjects. Our findings may not be directly applied to those patients with acute respiratory failure, it still provides an important reference/guidance for future studies. The investigation on the effects of utilizing different gas flows to deliver inhaled medication for patients with acute respiratory failure is demanded, particularly their long-term outcomes, such as the need for respiratory support or length of hospital stay, etc. Secondly, patients with COPD and asthma responded to bronchodilators at different doses, future studies are needed to investigate the effective dose and responses for two patient populations separately. Thirdly, unlike the robust ATS/ERS positive response criteria for identifying response to bronchodilators, the second criterion we applied has not been validated but is intended to identify the physiologic situation after standard bronchodilator therapy by monitoring FEV_1_ change greater than or equal to the screening levels. It should be noted that this criterion is not ideal, however, the variation in patients’ baseline situation prior to bronchodilator administration is hard to control. Future studies with a larger sample size are needed to confirm our findings, using ATS/ERS positive response criteria. Lastly, we only evaluated bronchodilator delivery, future studies are needed to investigate other inhaled medication, such as inhaled antibiotics or steroids.

## 5. Conclusions

During HFNC in which gas flow met or exceeded inspiratory flow, cumulative salbutamol doses of 1.5 mg and 3.5 mg produced substantial bronchodilator response across the groups, suggesting that standard unit doses of salbutamol might prove an effective starting dose for patients receiving HFNC. Applying the robust ATS/ERS criteria, no difference was observed among different flows, however, using the more sensitive criteria of subjects reaching post-screening FEV_1_ to salbutamol via HFNC, a higher number of subjects responded to the doses of 0.5 mg and 1.5 mg when HFNC gas flow was set at 50% of patient peak inspiratory flow.

## Figures and Tables

**Table 1:** Demographic information of patients in the three groups.

Patient Information	GF:IF = 0.5 (*n* = 25)	GF:IF = 1.0 (*n* = 24)	GF = 50 L/min (*n* = 26)	*p*
Age, years	51.2 ± 13.4	51.9 ± 16.6	51.1 ± 14.0	0.978
Male, %	16 (64%)	14 (58%)	18 (69%)	0.725
Asthma, %	16 (64%)	16 (67%)	17 (65%)	0.981
COPD, %	9 (36%)	8 (33%)	9 (35%)	
Height, cm	164.3 ± 6.8	166.2 ± 7.4	164.4 ± 7.7	0.587
Weight, Kg	71.7 ± 11.3	72.2 ± 12.8	68.7 ± 11.0	0.510
BMI, Kg/m^2^	26.5 ± 3.5	26.0 ± 3.3	25.4 ± 3.6	0.523
Vt, mL	766.4 ± 146.0	740.0 ± 190.3	800.2 ± 215.7	0.519
Peak inspiratory flow during tidal breathing, L/min	37.4 ± 7.8	34.5 ± 6.9	38.2 ± 6.0	0.137
HFNC flow settings, L/min	18.7 ± 3.9	34.5 ± 6.9	50	<0.001
Smoker, %	11 (44%)	11 (46%)	11 (42%)	0.969

GF, gas flow; IF, peak inspiratory flow during tidal breathing; COPD, chronic obstructive pulmonary disease; BMI, body mass index; Vt, tidal volume; HFNC, high-flow nasal cannula.

**Table 2:** Bronchodilation responses after inhaling salbutamol via HFNC among three groups.

Number of Patients (%)		Cumulative Dose	GF: IF = 0.5 (*n* = 25)	GF: IF = 1.0 (*n* = 24)	GF = 50 L/min (*n* = 26)	*p*
Criteria for bronchodilation responses	FEV_1_ increased by 200 mL and 12%	Saline	1 (4%)	0	1 (4%)	NA
		0.5 mg	11 (44%)	6 (25%)	7 (27%)	0.286
		1.5 mg	16 (64%)	14 (58%)	11 (42%)	0.271
		3.5 mg	17 (68%)	18 (75%)	18 (69%)	0.848
	FEV_1_ via HFNC ≥ FEV_1_ post MDI + Spacer	Saline	1 (4%)	0	0	NA
		0.5 mg	4 (16%)	3 (13%)	4 (15%)	0.934
		1.5 mg	16 (64%)	7 (29%)	7 (27%)	0.011
		3.5 mg	22 (88%)	11 (46%)	14 (54%)	0.005
Met either of the two criteria of FEV_1_		0.5 mg	13 (52%)	8 (33%)	10 (39%)	0.388
		1.5 mg	19 (76%)	17 (71%)	14 (54%)	0.213
		3.5 mg	23 (92%)	20 (83%)	21 (81%)	0.497
Met both criteria of FEV_1_		0.5 mg	2 (8%)	1 (4%)	1 (4%)	NA
		1.5 mg	13 (52%)	4 (17%)	4 (15%)	0.013
		3.5 mg	16 (64%)	9 (38%)	11 (42%)	0.366
FVC increased by 200 mL and 12%		0.5 mg	8 (32%)	5 (20%)	5 (19%)	0.513
		1.5 mg	13 (52%)	8 (33%)	10 (39%)	0.388
		3.5 mg	13 (52%)	10 (42%)	15 (58%)	0.520

GF, gas flow; IF, patient inspiratory flow; HFNC, high-flow nasal cannula; FEV_1_, forced expiratory volume in the first second; MDI, metered dose inhaler; VHC, valved holding chamber; ATS/ERS positive criteria: FEV_1_ increased by 12% and absolute volume increased ≥200 mL; ATS, American thoracic society; ERS, European respiratory society.

**Table 3:** The changes of FEV_1_ after inhaling salbutamol via MDI with VHC and after inhaling saline and salbutamol at different doses via HFNC.

Inhalation Device	FEV_1_ Changes	GF:IF = 0.5 (*n* = 25)	GF:IF = 1.0 (*n* = 24)	GF = 50 L/min (*n* = 26)	*p*
FEV_1_ (L) salbutamol (400 mcg) via MDI + VHC	Pre	1.65 ± 0.79	1.98 ± 0.80	1.91 ± 0.80	0.321 ^a^
	Pre FEV_1_ in predicted (%)	56.5 ± 23.8	66.2 ± 18.1	64.0 ± 18.9	0.221 ^a^
	Post	2.03 ± 0.83	2.34 ± 0.86	2.30 ± 0.87	0.577 ^a^
	Increase	0.375 ± 0.125	0.365 ± 0.110	0.389 ± 0.135	0.577 ^a^
	Increase (%)	27.3 ± 13.2	20.7 ± 9.3	23.5 ± 12.2	0.429 ^a^
FEV_1_ (L) with saline and salbutamol via VMN + HFNC	Pre	1.72 ± 0.84	1.91 ± 0.79	1.87 ± 0.80	0.691 ^a^
	Pre FEV_1_ in predicted (%)	58.5 ± 24.7	64.0 ± 19.0	63.2 ± 19.7	0.613 ^a^
	Saline	1.74 ± 0.87	1.90 ± 0.77	1.83 ± 0.81	0.060 ^b^
	0.5 mg	1.95 ± 0.86	2.10 ± 0.80	2.02 ± 0.81	0.194 ^b^
	1.5 mg	2.05 ± 0.86	2.20 ± 0.81	2.16 ± 0.84	0.804 ^b^
	3.5 mg	2.09 ± 0.87	2.28 ± 0.80	2.26 ± 0.84	0.968 ^b^
	7.5 mg ^c^	2.20 ± 0.93	2.21 ± 0.95	2.35 ± 0.89	0.567 ^b^
FEV_1_ increment (ml) with saline and salbutamol via VMN + HFNC	Saline	23 ± 87	−5 ± 65	−47 ± 139	0.060 ^b^
	0.5 mg	228 ± 146	197 ± 148	152 ± 147	0.194 ^b^
	1.5 mg	321 ± 161	298 ± 191	284 ± 240	0.804 ^b^
	3.5 mg	373 ± 171	375 ± 215	387 ± 264	0.968 ^b^
FEV_1_ increment (%) with saline and salbutamol via VMN + HFNC	Saline	0.7 ± 7.5	0 ± 5.0	−3.0 ± 8.5	0.140 ^b^
	0.5 mg	16.5 ± 14.0	12.1 ± 9.4	9.1 ± 9.4	0.087 ^b^
	1.5 mg	23.7 ± 17.0	17.7 ± 11.7	16.8 ± 14.4	0.283 ^b^
	3.5 mg	27.1 ± 18.2	23.2 ± 14.8	23.3 ± 16.0	0.831 ^b^

GF, gas flow; IF, patient inspiratory flow; HFNC, high-flow nasal cannula; FEV_1_, forced expiratory volume in the first second; MDI, metered dose inhaler; VHC, valved holding chamber. ^a^ comparison was conducted using ANOVA test; ^b^ comparison was conducted using ANCOVA test; ^c^ data available in 21, 21 and 23 patients in the three groups, respectively.

## Data Availability

Data are available upon reasonable request. Proposals should be directed to the corresponding author.

## Supplementary Materials

The following are available online at https://www.mdpi.com/article/10.3390/pharmaceutics13101655/s1, Table S1, Bronchodilation responses after inhaling salbutamol via HFNC among three groups for asthma and COPD patients; Table S2, The comparisons of FEV_1_ changes after inhaling saline and salbutamol via HFNC at different accumulative doses in three groups.
