# Supplementary material for: Bronchodilator Delivery via High-Flow Nasal Cannula: A Randomized Controlled Trial to Compare the Effects of Gas Flows
Source: Pharmaceutics. 2021 Oct 11;13(10):1655. doi: 10.3390/pharmaceutics13101655 (PMC8539308; doi:10.3390/pharmaceutics13101655)
Supplement: Supplementary file 1 [file pharmaceutics-13-01655-s001.zip › pharmaceutics-1361899-supplementary.pdf]

pharmaceutics

MDPI

Supplementary Materials: Bronchodilator Delivery via High-Flow Nasal Cannula: A Randomized Controlled Trial to Compare the Effects of Gas Flows

Jie Li, Yibing Chen, Stephan Ehrmann, Jie Wu, Lixin Xie and James B Fink

Table S1. Bronchodilation responses after inhaling salbutamol via HFNC among three groups for asthma and COPD patients.

			GF: IF = 0.5	GF: IF = 1.0	GF = 50 L/min	*p*
Asthma	No. of patients		16	16	17	
	Met ATS/ERS positive criteria, %	0.5mg	7 (43.8%)	6 (37.5%)	7 (41.2%)	0.937
		1.5mg	10 (62.5%)	9 (56.3%)	9 (52.9%)	0.854
		3.5mg	11 (68.8%)	11 (68.8%)	12 (70.6%)	0.991
	Post-bronchodilator FEV_1 via HFNC returns to Screening post-bronchodilator FEV_1	0.5mg	1 (6.3%)	1 (6.3%)	2 (11.8%)	0.798
		1.5mg	9 (56.3%)	3 (18.8%)	4 (23.5%)	0.047
		3.5mg	15 (93.8%)	6 (37.5%)	8 (47.1%)	0.002
COPD	No. of patients		9	8	9	
	Met ATS/ERS positive criteria, %	0.5mg	4 (44.4%)	0	0	0.012
		1.5mg	6 (66.7%)	5 (62.5%)	2 (22.2%)	0.118
		3.5mg	6 (66.7%)	7 (87.5%)	6 (66.7%)	0.848
	Post-bronchodilator FEV_1 via HFNC returns to screening post-bronchodilator FEV_1	0.5mg	3 (33.3%)	2 (25%)	2 (22.2%)	0.859
		1.5mg	7 (77.8%)	4 (50%)	3 (33.3%)	0.162
		3.5mg	7 (77.8%)	5 (62.5%)	6 (66.7%)	0.776

GF, gas flow; IF, patient inspiratory flow; HFNC, high-flow nasal cannula; FEV_1, forced expiratory volume at the first second; Screening, 400 mcg salbutamol via metered dose and valved holding chamber; ATS/ERS positive criteria: FEV_1 increased by 12% and absolute volume increased ≥ 200mL; COPD, chronic obstructive pulmonary disease.

Table S2. The comparisons of FEV_1 changes after inhaling saline and salbutamol via HFNC at different accumulative doses in three groups.

			GF:IF = 0.5	GF:IF = 1.0	GF = 50L/min	*p*
Asthma	No. of patients		16	16	17	
	FEV_1 Screening with Salbutamol	Pre	1.97 ± 0.76	2.18 ± .86	2.24 ± .70	
		Post	2.38 ± 0.78	2.58 ± .91	2.68 ± .74	
		Increase (ml)	411 ± 135	406 ± 109	443 ± 129	
		Increase (%)	24.1 ± 12.1	21.5 ± 10.6	21.7 ± 10.0	
	FEV_1 (L) via HFNC	Pre	2.04 ± 0.84	2.08 ± .85	2.15 ± .78	0.923
		NS	2.07 ± 0.87	2.07 ± .83	2.11 ± .79	0.317
		0.5mg	2.28 ± 0.86	2.31 ± .83	2.33 ± .76	0.572
		1.5mg	2.38 ± 0.84	2.42 ± .83	2.51 ± .74	0.940

		3.5mg	2.45 ± 0.82	2.50 ± .81	2.62 ± .73	0.746
		7.5mg [a]	2.60 ± 0.92	2.55 ± .89	2.73 ± .78	0.415
	FEV_1 increase (ml)	NS	34 ± 79	−4 ± 68	-41 ± 162	0.180
		0.5mg	238 ± 157	236 ± 165	181 ± 170	0.572
		1.5mg	343 ± 170	349 ± 208	361 ± 254	0.940
		3.5mg	413 ± 170	424 ± 245	464 ± 292	0.746
	FEV_1 increase (%)	0.5mg	14.6 ± 14.9	13.6 ± 10.4	9.9 ± 10.7	0.545
		1.5mg	21.4 ± 17.4	19.5 ± 13.0	19.9 ± 15.9	0.936
		3.5mg	26.1 ± 20.0	24.8 ± 17.0	25.5 ± 18.9	0.925
COPD	No. of patients		9	8	9	
	FEV_1 (ml) Screening with Salbutamol	Pre	1.09 ± 0.47	1.58 ± 0.48	1.28 ± 0.58	
		Post	1.40 ± 0.48	1.87 ± 0.51	1.57 ± 0.60	
		Increase (mL)	310 ± 73	284 ± 52	288 ± 76	
		Increase (%)	32.9 ± 14.0	19.3 ± 6.5	27.0 ± 15.7	
	FEV_1 (L) via HFNC	Pre	1.15 ± 0.47	1.58 ± 0.57	1.34 ± 0.53	0.278
		NS	1.15 ± 0.49	1.56 ± 0.55	1.28 ± 0.53	0.180
		0.5mg	1.36 ± 0.50	1.69 ± 0.56	1.44 ± 0.55	0.040
		1.5mg	1.43 ± 0.49	1.76 ± 0.58	1.48 ± 0.57	0.060
		3.5mg	1.45 ± 0.51	1.84 ± 0.58	1.58 ± 0.58	0.505
		7.5mg [b]	1.55 ± 0.48	1.75 ± 0.41	1.66 ± 0.65	0.885
	FEV_1 increase (ml) from Baseline	NS	2 ± 102	−5 ± 65	-57 ± 87	0.317
		0.5mg	210 ± 132	119 ± 52	97 ± 69	0.040
		1.5mg	282 ± 143	195 ± 97	138 ± 120	0.060
		3.5mg	301 ± 156	276 ± 81	242 ± 104	0.505
	FEV_1 increase (%) from Baseline	0.5mg	19.8 ± 12.4	9.0 ± 6.4	7.7 ± 6.8	0.042
		1.5mg	27.7 ± 16.6	14.1 ± 8.4	11.0 ± 9.4	0.036
		3.5mg	29.0 ± 15.5	20.0 ± 9.4	19.0 ± 7.9	0.287

GF, gas flow; IF, patient inspiratory flow; HFNC, high-flow nasal cannula; FEV_1, forced expiratory volume at the first second; Screening, 400 mcg salbutamol via metered dose inhaler and valved holding chamber; ATS/ERS positive criteria: FEV_1 increased by 12% and absolute volume increased ≥ 200mL; COPD, chronic obstructive pulmonary disease. [a] Data was available in 13,14, and 15 patients, respectively; [b] data was available in 8,7 and 8 patients, respectively.
